# 1,1′,2,2′-Tetra­methyl-3,3′-(4-methoxy­benzyl­idene)di-1*H*-indole

**DOI:** 10.1107/S1600536809017759

**Published:** 2009-05-20

**Authors:** Cai-Li Zhang, Ping-Ping Ye, Zhi-Qiang Du

**Affiliations:** a Department of Chemistry, Zhejiang University, Hangzhou 310027, People’s Republic of China

## Abstract

The title compound, C_28_H_28_N_2_O, was prepared by condensation of 1,2-bimethyl­indole and 4-methoxy­benzaldehyde. In the mol­ecular structure, the plane of the non-fused benzene ring is twisted with respect to the planes of the two indole ring systems, exhibiting dihedral angles of 72.04 (7) and 72.24 (7)°, while the planes of the two indole ring systems are oriented at a dihedral angle of 87.05 (5)°. Neither hydrogen bonding nor π–π stacking is observed in the crystal structure.

## Related literature

For general background to the physiological properties of indole derivatives, see: Poter *et al.* (1977 ▶); Sundberg (1996 ▶). For related structures, see: Chang *et al.* (1999 ▶); Ge *et al.* (1999 ▶); Morris & Andersen (1990 ▶); Azizian *et al.* (2007 ▶); Osawa & Namiki (1983 ▶). For the synthesis, see: Deb & Bhuyan (2006 ▶).
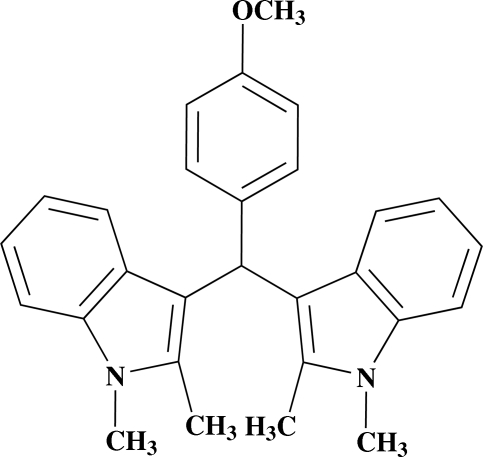


## Experimental

### 

#### Crystal data

C_28_H_28_N_2_O
*M* *_r_* = 408.52
Monoclinic, 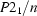

*a* = 10.6647 (8) Å
*b* = 13.2088 (10) Å
*c* = 16.1494 (13) Å
β = 97.1740 (10)°
*V* = 2257.1 (3) Å^3^
*Z* = 4
Mo *K*α radiation
μ = 0.07 mm^−1^
*T* = 296 K
0.25 × 0.24 × 0.21 mm

#### Data collection

Bruker SMART CCD area-detector diffractometer
Absorption correction: none
11505 measured reflections
3955 independent reflections
3196 reflections with *I* > 2σ(*I*)
*R*_int_ = 0.025

#### Refinement

*R*[*F* ^2^ > 2σ(*F* ^2^)] = 0.044
*wR*(*F* ^2^) = 0.139
*S* = 0.83
3955 reflections
285 parameters
H-atom parameters constrained
Δρ_max_ = 0.21 e Å^−3^
Δρ_min_ = −0.22 e Å^−3^

### 

Data collection: *SMART* (Bruker, 2004 ▶); cell refinement: *SAINT* (Bruker, 2004 ▶); data reduction: *SAINT*; program(s) used to solve structure: *SHELXS97* (Sheldrick, 2008 ▶); program(s) used to refine structure: *SHELXL97* (Sheldrick, 2008 ▶); molecular graphics: *ORTEP-3 for Windows* (Farrugia, 1997 ▶); software used to prepare material for publication: *WinGX* (Farrugia, 1999 ▶).

## Supplementary Material

Crystal structure: contains datablocks I, global. DOI: 10.1107/S1600536809017759/xu2523sup1.cif

Structure factors: contains datablocks I. DOI: 10.1107/S1600536809017759/xu2523Isup2.hkl

Additional supplementary materials:  crystallographic information; 3D view; checkCIF report

## Figures and Tables

**Fig. 1.**
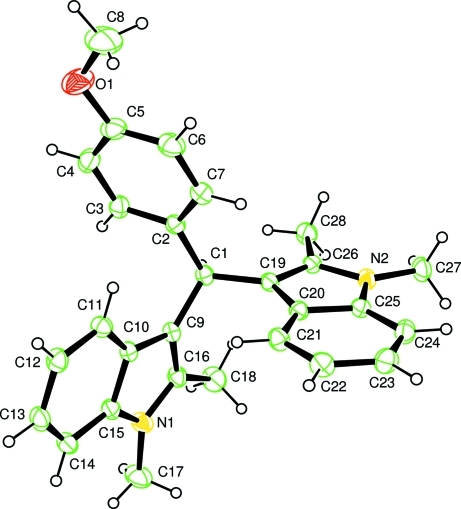
The molecular structure of (I) with 30% probability displacement ellipsoids.

## supplementary crystallographic information

### Comment

Indole derivatives are found abundantly in a variety of natural plants and exhibit various physiological properties (Poter *et al.*, 1977; Sundberg, 1996). Among them, bis-indolymethane derivatives are found to be kinds of potentially bioactive compounds (Chang *et al.*, 1999; Ge *et al.*, 1999). In recent years, the synthesis and application of bis-indolymethane derivatives have been widely studied (Osawa & Namiki, 1983; Morris & Andersen, 1990; Azizian *et al.*, 2007). The title compound is one of bis-indolymethane derivatives. We report here its crystal structure.

The molecular structure of the title compound is shown in Fig. 1. The C2-benzene ring is twisted to the two indole rings with the dihedral angles of 72.04 (7)° and 72.24 (7)°, respectively. Two indole rings make a dihedral angle of 87.86 (3)° to each other. Neither hydrogen bonding nor π-π stacking is observed in the crystal structure.

### Experimental

The title compound was prepared according to the procedure reported by Deb & Bhuyan (2006). 1,2-Bimethylindole (2.0 mmol) and *p*-methoxybenzaldehyde (1.0 mmol) were dissolved in methanol (5 ml). The solution was stirred at room temperature for 5 h, then the solvent was removed under vacuum and the crude product was purified by silica-gel flash column chromatography. Single crystals suitable for X-ray data collection were obtained by recrystallization from chloroform/hexane.

### Refinement

H atoms were located geometrically and treated as riding, with C—H = 0.93 (aromatic), 0.96 (methyl) and 0.98 Å (methine), and *U*_iso_(H) = 1.5*U*_eq_(C) for methyl and 1.2U_eq_(C) for the others.

### Crystal data

**Table:**

C_28_H_28_N_2_O	*F*(000) = 872
*M**_r_* = 408.52	*D*_x_ = 1.202 Mg m^−^^3^
Monoclinic, *P*2_1_/*n*	Mo *K*α radiation, λ = 0.71073 Å
Hall symbol: -P 2yn	Cell parameters from 6354 reflections
*a* = 10.6647 (8) Å	θ = 4.9–51.2°
*b* = 13.2088 (10) Å	µ = 0.07 mm^−^^1^
*c* = 16.1494 (13) Å	*T* = 296 K
β = 97.174 (1)°	Prism, colorless
*V* = 2257.1 (3) Å^3^	0.25 × 0.24 × 0.21 mm
*Z* = 4	

### Data collection

**Table:**

Bruker SMART CCD area-detector diffractometer	3196 reflections with *I* > 2σ(*I*)
Radiation source: fine-focus sealed tube	*R*_int_ = 0.025
graphite	θ_max_ = 25.0°, θ_min_ = 2.0°
φ and ω scans	*h* = −12→12
11505 measured reflections	*k* = −15→15
3955 independent reflections	*l* = −19→19

### Refinement

**Table:**

Refinement on *F*^2^	Primary atom site location: structure-invariant direct methods
Least-squares matrix: full	Secondary atom site location: difference Fourier map
*R*[*F*^2^ > 2σ(*F*^2^)] = 0.044	Hydrogen site location: inferred from neighbouring sites
*wR*(*F*^2^) = 0.139	H-atom parameters constrained
*S* = 0.83	*w* = 1/[σ^2^(*F*_o_^2^) + (0.1047*P*)^2^ + 0.8752*P*] where *P* = (*F*_o_^2^ + 2*F*_c_^2^)/3
3955 reflections	(Δ/σ)_max_ < 0.001
285 parameters	Δρ_max_ = 0.21 e Å^−^^3^
0 restraints	Δρ_min_ = −0.22 e Å^−^^3^

### Special details

**Table:**

Geometry. All e.s.d.'s (except the e.s.d. in the dihedral angle between two l.s. planes) are estimated using the full covariance matrix. The cell e.s.d.'s are taken into account individually in the estimation of e.s.d.'s in distances, angles and torsion angles; correlations between e.s.d.'s in cell parameters are only used when they are defined by crystal symmetry. An approximate (isotropic) treatment of cell e.s.d.'s is used for estimating e.s.d.'s involving l.s. planes.
Refinement. Refinement of *F*^2^ against ALL reflections. The weighted *R*-factor *wR* and goodness of fit *S* are based on *F*^2^, conventional *R*-factors *R* are based on *F*, with *F* set to zero for negative *F*^2^. The threshold expression of *F*^2^ > σ(*F*^2^) is used only for calculating *R*-factors(gt) *etc*. and is not relevant to the choice of reflections for refinement. *R*-factors based on *F*^2^ are statistically about twice as large as those based on *F*, and *R*- factors based on ALL data will be even larger.

### Fractional atomic coordinates and isotropic or equivalent isotropic displacement parameters (Å^2^)

**Table:**

	*x*	*y*	*z*	*U*_iso_*/*U*_eq_	
N1	0.63286 (14)	0.32330 (11)	0.89904 (8)	0.0421 (4)	
N2	0.23060 (12)	0.47645 (10)	0.62297 (8)	0.0358 (3)	
O1	0.73808 (18)	0.09952 (13)	0.43460 (10)	0.0827 (5)	
C1	0.54764 (14)	0.35901 (11)	0.67122 (9)	0.0316 (3)	
H1	0.5937	0.4226	0.6671	0.038*	
C2	0.59177 (14)	0.28915 (11)	0.60516 (9)	0.0334 (4)	
C3	0.72045 (16)	0.27569 (12)	0.60290 (11)	0.0403 (4)	
H3	0.7776	0.3097	0.6414	0.048*	
C4	0.76556 (18)	0.21341 (14)	0.54521 (12)	0.0501 (5)	
H4	0.8522	0.2066	0.5446	0.060*	
C5	0.6831 (2)	0.16106 (14)	0.48826 (12)	0.0529 (5)	
C6	0.5550 (2)	0.17324 (15)	0.48860 (12)	0.0544 (5)	
H6	0.4984	0.1387	0.4502	0.065*	
C7	0.51073 (17)	0.23765 (13)	0.54694 (11)	0.0435 (4)	
H7	0.4240	0.2460	0.5464	0.052*	
C8	0.6598 (4)	0.0526 (3)	0.36972 (17)	0.1146 (13)	
H8A	0.7107	0.0159	0.3349	0.172*	
H8B	0.6113	0.1031	0.3371	0.172*	
H8C	0.6036	0.0066	0.3927	0.172*	
C9	0.58732 (14)	0.32027 (11)	0.75930 (9)	0.0319 (3)	
C10	0.62303 (14)	0.21961 (11)	0.78731 (10)	0.0317 (3)	
C11	0.63396 (15)	0.12468 (12)	0.74924 (10)	0.0373 (4)	
H11	0.6147	0.1177	0.6918	0.045*	
C12	0.67336 (16)	0.04226 (12)	0.79772 (11)	0.0423 (4)	
H12	0.6806	−0.0203	0.7724	0.051*	
C13	0.70262 (16)	0.05046 (13)	0.88377 (11)	0.0446 (4)	
H13	0.7297	−0.0065	0.9148	0.054*	
C14	0.69222 (16)	0.14123 (14)	0.92376 (11)	0.0428 (4)	
H14	0.7117	0.1467	0.9813	0.051*	
C15	0.65141 (14)	0.22480 (12)	0.87502 (10)	0.0355 (4)	
C16	0.59485 (15)	0.38016 (12)	0.82871 (10)	0.0377 (4)	
C17	0.6468 (2)	0.35910 (17)	0.98459 (12)	0.0627 (6)	
H17A	0.6664	0.3030	1.0218	0.094*	
H17B	0.5693	0.3901	0.9960	0.094*	
H17C	0.7139	0.4079	0.9926	0.094*	
C18	0.5666 (2)	0.49042 (14)	0.83534 (13)	0.0561 (5)	
H18A	0.6390	0.5245	0.8638	0.084*	
H18B	0.4958	0.4992	0.8660	0.084*	
H18C	0.5467	0.5185	0.7804	0.084*	
C19	0.40945 (14)	0.38686 (11)	0.65659 (9)	0.0314 (3)	
C20	0.30467 (14)	0.32631 (11)	0.67439 (9)	0.0325 (4)	
C21	0.29216 (16)	0.22881 (12)	0.70659 (10)	0.0395 (4)	
H21	0.3633	0.1891	0.7218	0.047*	
C22	0.17413 (17)	0.19248 (14)	0.71551 (11)	0.0455 (4)	
H22	0.1659	0.1277	0.7368	0.055*	
C23	0.06594 (17)	0.25115 (15)	0.69312 (12)	0.0484 (4)	
H23	−0.0130	0.2249	0.7000	0.058*	
C24	0.07467 (16)	0.34710 (14)	0.66114 (11)	0.0438 (4)	
H24	0.0028	0.3859	0.6458	0.053*	
C25	0.19443 (15)	0.38423 (12)	0.65254 (9)	0.0339 (4)	
C26	0.36095 (14)	0.47703 (11)	0.62486 (9)	0.0322 (3)	
C27	0.14395 (17)	0.55850 (14)	0.59566 (12)	0.0477 (4)	
H27A	0.0825	0.5654	0.6340	0.072*	
H27B	0.1018	0.5437	0.5410	0.072*	
H27C	0.1904	0.6206	0.5940	0.072*	
C28	0.42723 (17)	0.56692 (13)	0.59485 (11)	0.0440 (4)	
H28A	0.4049	0.6263	0.6240	0.066*	
H28B	0.4025	0.5756	0.5361	0.066*	
H28C	0.5169	0.5566	0.6051	0.066*	

### Atomic displacement parameters (Å^2^)

**Table:**

	*U*^11^	*U*^22^	*U*^33^	*U*^12^	*U*^13^	*U*^23^
N1	0.0508 (8)	0.0427 (8)	0.0313 (7)	0.0060 (6)	−0.0012 (6)	−0.0044 (6)
N2	0.0330 (7)	0.0350 (7)	0.0386 (7)	0.0063 (5)	0.0018 (6)	0.0039 (6)
O1	0.1152 (14)	0.0747 (11)	0.0624 (10)	0.0308 (10)	0.0278 (9)	−0.0145 (8)
C1	0.0315 (8)	0.0275 (8)	0.0353 (8)	−0.0008 (6)	0.0018 (6)	0.0037 (6)
C2	0.0382 (9)	0.0299 (8)	0.0324 (8)	0.0016 (6)	0.0052 (6)	0.0063 (6)
C3	0.0371 (9)	0.0382 (9)	0.0457 (10)	0.0028 (7)	0.0058 (7)	0.0039 (7)
C4	0.0499 (11)	0.0477 (11)	0.0557 (11)	0.0115 (8)	0.0177 (9)	0.0075 (9)
C5	0.0758 (14)	0.0434 (10)	0.0417 (10)	0.0161 (9)	0.0160 (9)	0.0026 (8)
C6	0.0690 (13)	0.0507 (11)	0.0416 (10)	0.0012 (9)	−0.0004 (9)	−0.0076 (8)
C7	0.0432 (9)	0.0474 (10)	0.0390 (9)	0.0005 (7)	0.0012 (7)	−0.0003 (8)
C8	0.173 (3)	0.112 (2)	0.0558 (15)	0.059 (2)	0.0022 (18)	−0.0304 (16)
C9	0.0294 (7)	0.0305 (8)	0.0347 (8)	0.0012 (6)	−0.0007 (6)	0.0008 (6)
C10	0.0281 (7)	0.0323 (8)	0.0339 (8)	0.0007 (6)	0.0008 (6)	0.0018 (6)
C11	0.0414 (9)	0.0336 (8)	0.0366 (9)	0.0005 (7)	0.0037 (7)	0.0017 (7)
C12	0.0446 (9)	0.0316 (8)	0.0514 (10)	0.0054 (7)	0.0089 (8)	0.0044 (7)
C13	0.0410 (9)	0.0411 (10)	0.0516 (11)	0.0064 (7)	0.0047 (8)	0.0156 (8)
C14	0.0398 (9)	0.0508 (10)	0.0366 (9)	0.0029 (8)	−0.0005 (7)	0.0085 (8)
C15	0.0337 (8)	0.0380 (9)	0.0338 (8)	0.0024 (7)	0.0001 (6)	0.0012 (7)
C16	0.0391 (9)	0.0338 (9)	0.0391 (9)	0.0042 (7)	0.0007 (7)	−0.0020 (7)
C17	0.0839 (15)	0.0651 (13)	0.0362 (10)	0.0102 (11)	−0.0041 (10)	−0.0105 (9)
C18	0.0727 (14)	0.0391 (10)	0.0550 (12)	0.0119 (9)	0.0018 (10)	−0.0095 (9)
C19	0.0324 (8)	0.0320 (8)	0.0294 (8)	0.0011 (6)	0.0024 (6)	0.0019 (6)
C20	0.0337 (8)	0.0337 (8)	0.0295 (8)	0.0006 (6)	0.0020 (6)	0.0000 (6)
C21	0.0385 (9)	0.0373 (9)	0.0416 (9)	−0.0008 (7)	0.0011 (7)	0.0051 (7)
C22	0.0453 (10)	0.0423 (10)	0.0485 (10)	−0.0090 (8)	0.0048 (8)	0.0065 (8)
C23	0.0375 (9)	0.0545 (11)	0.0536 (11)	−0.0099 (8)	0.0079 (8)	0.0008 (9)
C24	0.0338 (9)	0.0490 (10)	0.0481 (10)	0.0024 (7)	0.0030 (7)	−0.0022 (8)
C25	0.0340 (8)	0.0365 (8)	0.0309 (8)	0.0016 (6)	0.0025 (6)	−0.0013 (6)
C26	0.0335 (8)	0.0326 (8)	0.0305 (8)	0.0023 (6)	0.0036 (6)	0.0012 (6)
C27	0.0435 (10)	0.0446 (10)	0.0535 (11)	0.0141 (8)	0.0003 (8)	0.0048 (8)
C28	0.0461 (10)	0.0368 (9)	0.0492 (10)	0.0022 (7)	0.0069 (8)	0.0103 (7)

### Geometric parameters (Å, °)

**Table:**

N1—C15	1.379 (2)	C12—H12	0.9300
N1—C16	1.380 (2)	C13—C14	1.373 (3)
N1—C17	1.450 (2)	C13—H13	0.9300
N2—C25	1.381 (2)	C14—C15	1.394 (2)
N2—C26	1.3867 (19)	C14—H14	0.9300
N2—C27	1.457 (2)	C16—C18	1.494 (2)
O1—C5	1.372 (2)	C17—H17A	0.9600
O1—C8	1.401 (4)	C17—H17B	0.9600
C1—C19	1.509 (2)	C17—H17C	0.9600
C1—C9	1.521 (2)	C18—H18A	0.9600
C1—C2	1.529 (2)	C18—H18B	0.9600
C1—H1	0.9800	C18—H18C	0.9600
C2—C7	1.375 (2)	C19—C26	1.372 (2)
C2—C3	1.389 (2)	C19—C20	1.432 (2)
C3—C4	1.374 (2)	C20—C21	1.401 (2)
C3—H3	0.9300	C20—C25	1.410 (2)
C4—C5	1.377 (3)	C21—C22	1.371 (2)
C4—H4	0.9300	C21—H21	0.9300
C5—C6	1.376 (3)	C22—C23	1.400 (3)
C6—C7	1.395 (3)	C22—H22	0.9300
C6—H6	0.9300	C23—C24	1.376 (3)
C7—H7	0.9300	C23—H23	0.9300
C8—H8A	0.9600	C24—C25	1.391 (2)
C8—H8B	0.9600	C24—H24	0.9300
C8—H8C	0.9600	C26—C28	1.493 (2)
C9—C16	1.366 (2)	C27—H27A	0.9600
C9—C10	1.440 (2)	C27—H27B	0.9600
C10—C11	1.408 (2)	C27—H27C	0.9600
C10—C15	1.413 (2)	C28—H28A	0.9600
C11—C12	1.376 (2)	C28—H28B	0.9600
C11—H11	0.9300	C28—H28C	0.9600
C12—C13	1.390 (3)		
			
C15—N1—C16	108.65 (13)	N1—C15—C14	129.36 (15)
C15—N1—C17	125.05 (15)	N1—C15—C10	107.91 (13)
C16—N1—C17	126.26 (15)	C14—C15—C10	122.72 (15)
C25—N2—C26	108.65 (12)	C9—C16—N1	109.97 (14)
C25—N2—C27	124.55 (14)	C9—C16—C18	129.17 (16)
C26—N2—C27	126.80 (14)	N1—C16—C18	120.86 (15)
C5—O1—C8	118.2 (2)	N1—C17—H17A	109.5
C19—C1—C9	112.38 (12)	N1—C17—H17B	109.5
C19—C1—C2	114.70 (13)	H17A—C17—H17B	109.5
C9—C1—C2	112.06 (12)	N1—C17—H17C	109.5
C19—C1—H1	105.6	H17A—C17—H17C	109.5
C9—C1—H1	105.6	H17B—C17—H17C	109.5
C2—C1—H1	105.6	C16—C18—H18A	109.5
C7—C2—C3	117.22 (15)	C16—C18—H18B	109.5
C7—C2—C1	123.63 (14)	H18A—C18—H18B	109.5
C3—C2—C1	119.15 (14)	C16—C18—H18C	109.5
C4—C3—C2	121.69 (17)	H18A—C18—H18C	109.5
C4—C3—H3	119.2	H18B—C18—H18C	109.5
C2—C3—H3	119.2	C26—C19—C20	107.00 (13)
C5—C4—C3	120.34 (18)	C26—C19—C1	125.84 (14)
C5—C4—H4	119.8	C20—C19—C1	127.15 (13)
C3—C4—H4	119.8	C21—C20—C25	118.45 (14)
O1—C5—C4	115.59 (19)	C21—C20—C19	134.46 (14)
O1—C5—C6	125.1 (2)	C25—C20—C19	107.09 (13)
C4—C5—C6	119.35 (17)	C22—C21—C20	119.45 (16)
C5—C6—C7	119.62 (18)	C22—C21—H21	120.3
C5—C6—H6	120.2	C20—C21—H21	120.3
C7—C6—H6	120.2	C21—C22—C23	121.13 (17)
C2—C7—C6	121.77 (17)	C21—C22—H22	119.4
C2—C7—H7	119.1	C23—C22—H22	119.4
C6—C7—H7	119.1	C24—C23—C22	120.98 (16)
O1—C8—H8A	109.5	C24—C23—H23	119.5
O1—C8—H8B	109.5	C22—C23—H23	119.5
H8A—C8—H8B	109.5	C23—C24—C25	117.91 (16)
O1—C8—H8C	109.5	C23—C24—H24	121.0
H8A—C8—H8C	109.5	C25—C24—H24	121.0
H8B—C8—H8C	109.5	N2—C25—C24	130.18 (15)
C16—C9—C10	106.89 (13)	N2—C25—C20	107.76 (13)
C16—C9—C1	123.61 (14)	C24—C25—C20	122.07 (15)
C10—C9—C1	129.50 (14)	C19—C26—N2	109.49 (13)
C11—C10—C15	117.53 (14)	C19—C26—C28	129.77 (14)
C11—C10—C9	135.90 (15)	N2—C26—C28	120.74 (13)
C15—C10—C9	106.57 (13)	N2—C27—H27A	109.5
C12—C11—C10	119.50 (15)	N2—C27—H27B	109.5
C12—C11—H11	120.2	H27A—C27—H27B	109.5
C10—C11—H11	120.2	N2—C27—H27C	109.5
C11—C12—C13	121.45 (16)	H27A—C27—H27C	109.5
C11—C12—H12	119.3	H27B—C27—H27C	109.5
C13—C12—H12	119.3	C26—C28—H28A	109.5
C14—C13—C12	121.14 (16)	C26—C28—H28B	109.5
C14—C13—H13	119.4	H28A—C28—H28B	109.5
C12—C13—H13	119.4	C26—C28—H28C	109.5
C13—C14—C15	117.65 (16)	H28A—C28—H28C	109.5
C13—C14—H14	121.2	H28B—C28—H28C	109.5
C15—C14—H14	121.2		
